# External Neurolysis and Hypothenar Fat Pad Flap With Early Nerve Gliding Exercise Regimen for Recurrent Carpal Tunnel Syndrome

**DOI:** 10.7759/cureus.91061

**Published:** 2025-08-26

**Authors:** Aidan J Maxwell, Ian Risser, Isaac A Arefi, Ahmed Suparno Bahar Moni

**Affiliations:** 1 Medicine, University of Toledo College of Medicine & Life Sciences, Toledo, USA; 2 Orthopedic Surgery, University of Toledo College of Medicine & Life Sciences, Toledo, USA

**Keywords:** carpal tunnel syndrome, entrapment neuropathy, external neurolysis, hypothenar fat pad flap, median nerve compression, nerve gliding exercises, peripheral neuropathy, recurrent cts, scar adhesion

## Abstract

Carpal tunnel syndrome (CTS) is the most common compressive neuropathy of the upper extremity and is most often treated successfully with surgical release of the median nerve. However, some patients continue to experience symptoms or develop recurrence, which presents a management challenge. The pathophysiology of recurrent CTS may involve perineural fibrosis, iatrogenic injury, incomplete release, or unrelated neuropathic conditions such as diabetic or hereditary neuropathies. Management depends on patient history, examination, and clinical factors, with many requiring revisional surgery to relieve compression and protect the nerve. External neurolysis and hypothenar fat pad flap procedures can release the median nerve while providing soft tissue coverage to reduce scar adhesion. This report presents two cases of recurrent CTS treated successfully with this approach, highlighting its potential value as a treatment option and the importance of meticulous surgical technique and postoperative care.

## Introduction

Carpal tunnel syndrome (CTS) is the most common peripheral neuropathy, caused by compression of the median nerve as it passes beneath the transverse carpal ligament (TCL) [1]. It affects an estimated 1%-5% of adults, with risk factors including repetitive hand use, obesity, diabetes, and other comorbidities [2]. In the United States, approximately 500,000 carpal tunnel release (CTR) procedures are performed each year [1,3,4]. CTR is the standard treatment when conservative measures fail, providing symptom resolution in 80%-95% of cases [3,5].

Recalcitrant CTS may present as persistent, recurrent, or new symptoms [6]. Persistent symptoms often result from incomplete TCL release, double-crush syndrome, misdiagnosis, or irreversible pathology [3,6]. Recurrent symptoms, commonly due to perineural fibrosis, follow an initial period of improvement [7,8]. New symptoms are defined as novel sensory or motor complaints arising after a previously successful CTR [6].

No standardized guidelines exist for managing recalcitrant CTS. Conservative options include splinting, physiotherapy, nerve gliding, and medications [3]. Surgical options include revision decompression, external or internal neurolysis, grafting, or tissue interposition [3,6]. An incomplete TCL release is best treated with open neurolysis [3,6]. Perineural adhesions, reported in up to 88% of revision cases [3], are typically managed with soft tissue coverage, such as venous or collagen wraps, muscle flaps, or hypothenar fat pad flaps [5,6]. The hypothenar fat pad flap offers a less invasive option using the original CTR incision and avoids the morbidity of more complex reconstructions. Studies suggest this technique improves symptom relief and function while minimizing complications such as flap tension or ischemia [5,6,9].

Here, we present two cases of recurrent CTS due to postoperative scarring, treated with external neurolysis and hypothenar fat pad flap, with excellent outcomes. We also describe the surgical technique, intraoperative findings, and an early nerve gliding rehabilitation protocol.

## Case presentation

Case 1 

A 68-year-old man presented with a three-year history of paresthesia in the thumb, index, and long fingers of the left hand. He had undergone CTR five years earlier with complete symptom resolution. Symptoms recurred one year postoperatively, initially limited to the radial digits, and later progressing to involve all fingers and the ulnar forearm. The CTS-6 score was 17.5. He was diagnosed with recurrent CTS and concurrent cubital tunnel syndrome (CuTS). Conservative measures, including wrist splinting, physiotherapy, and nerve-gliding exercises, were ineffective.

On examination, Phalen and Tinel signs were positive at the wrist, with reduced sensation in the median nerve distribution and mild thenar atrophy. Nerve conduction studies were within normal limits for both the median and ulnar nerves. Central nervous system disorders, polyneuropathy, hereditary neuropathy with liability to pressure palsies, and diabetic neuropathy were ruled out.

**Figure 1:**
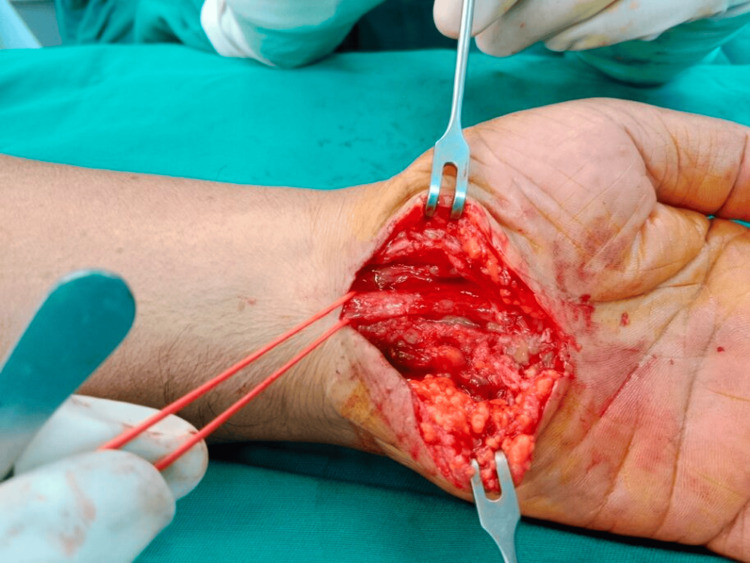
Intraoperative external neurolysis of the median nerve in a 68-year-old man (Case 1).

**Figure 2:**
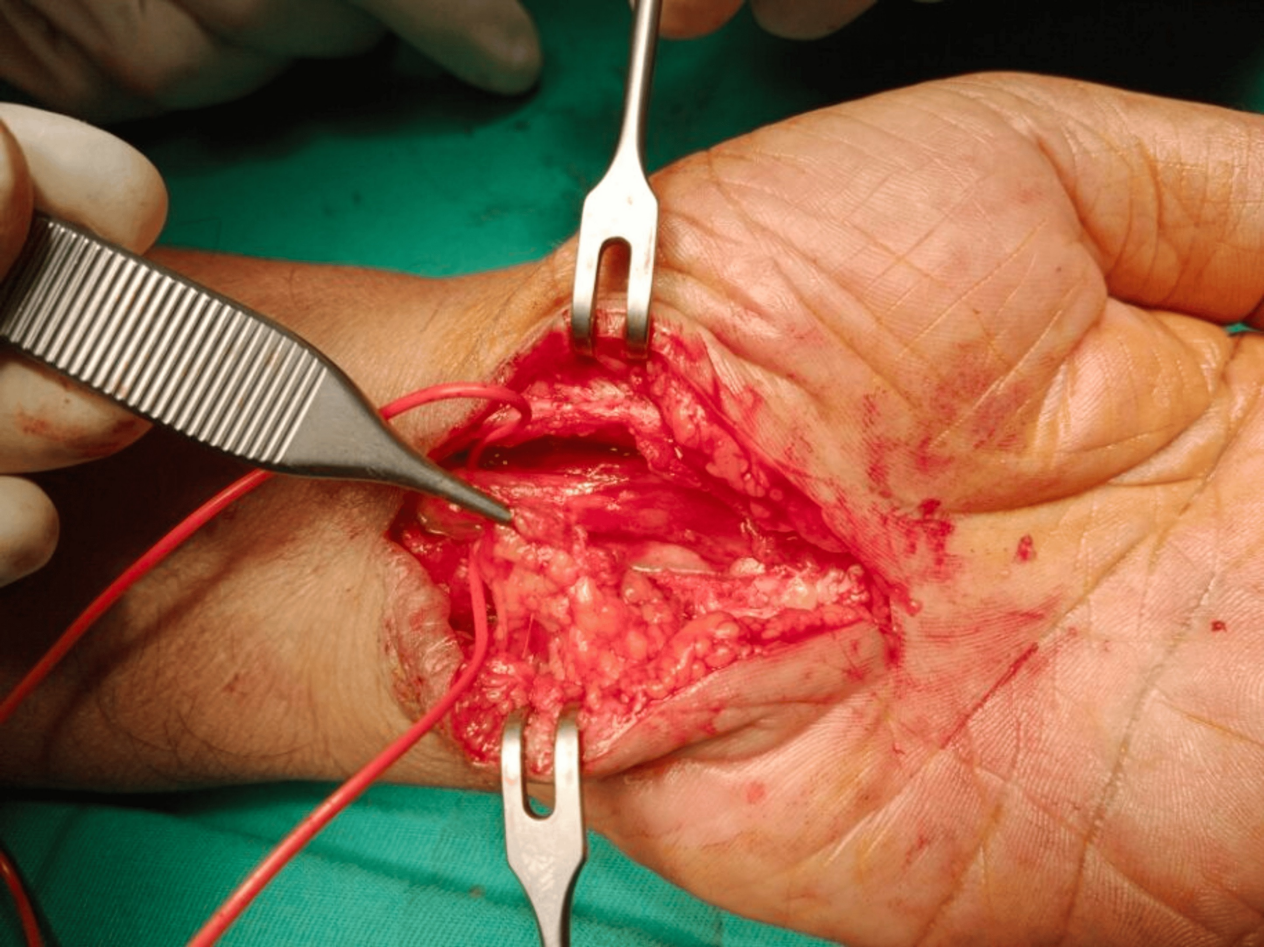
Hypothenar fat pad flap covering the neurolysed median nerve in a 68-year-old man (Case 1).

The patient underwent ulnar nerve decompression with anterior submuscular transposition, along with external neurolysis and hypothenar fat pad flap for median nerve coverage (Figure 1 and Figure 2). Intraoperatively, dense perineural adhesions were found along the proximal and distal median nerve. External neurolysis was performed to free the nerve from scar tissue, and a well-vascularized hypothenar fat pad flap was rotated and sutured to provide tension-free coverage. Flap positioning was confirmed by ensuring smooth nerve gliding without compression or angulation. At the three-month follow-up, symptoms in the median nerve distribution had completely resolved, while ulnar nerve symptoms persisted.

Case 2 

A 65-year-old woman presented with tingling and numbness in the radial three fingers of the right hand, along with thenar atrophy. She had previously undergone CTR 10 years earlier. The CTS-6 score was 13.5. Clinical history and nerve conduction studies confirmed recurrent CTS with concurrent CuTS. Conservative management was unsuccessful.

Preoperative examination showed positive Phalen and Tinel signs, sensory deficits in the median nerve distribution, and mild motor weakness. Other neuropathies and central disorders were excluded.

**Figure 3:**
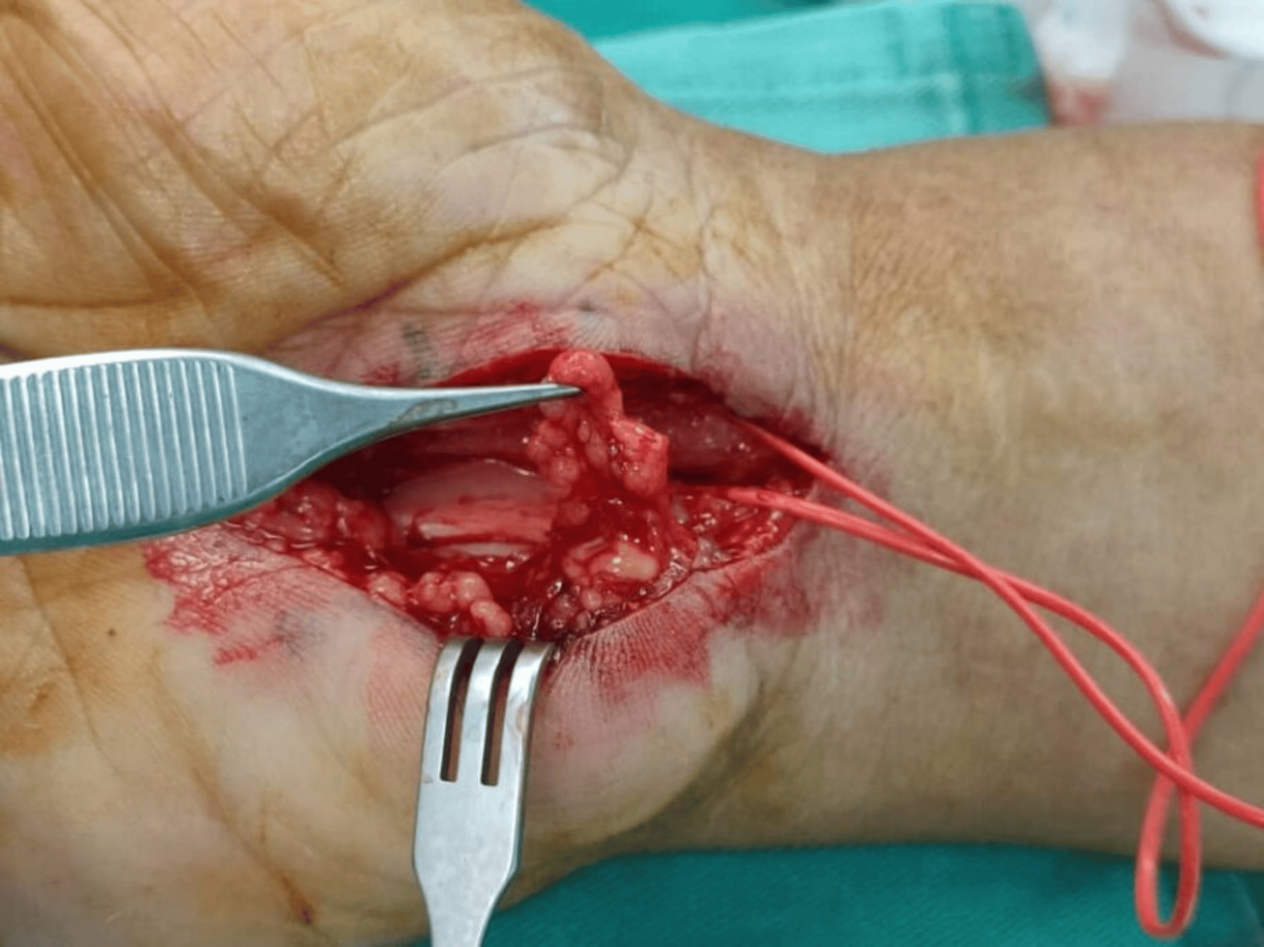
Intraoperative external neurolysis in a 65-year-old woman (Case 2).

**Figure 4:**
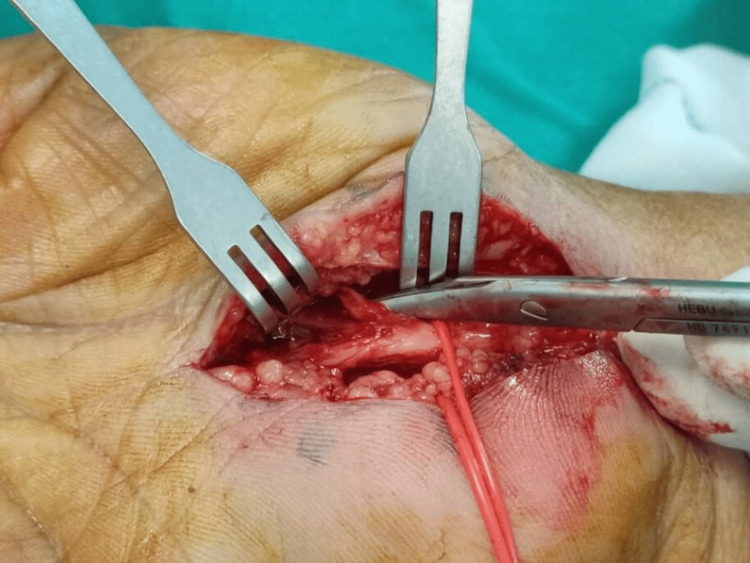
Hypothenar fat pad flap covering the median nerve post-neurolysis (Case 2).

Surgery consisted of ulnar nerve decompression with anterior submuscular transposition and external neurolysis with hypothenar fat pad flap (Figure 3 and Figure 4). Intraoperative findings demonstrated circumferential perineural scarring around the median nerve. The hypothenar fat pad flap was mobilized and sutured without tension, ensuring smooth nerve gliding.

## Discussion

CTS is the most common entrapment neuropathy, affecting 1%-5% of adults worldwide, and is effectively managed by surgical release of the TCL [1]. CTR is the standard treatment; however, a subset of patients develop persistent, recurrent, or new symptoms [6]. Persistent symptoms are often due to incomplete release, recurrent symptoms are usually related to perineural fibrosis, and new symptoms represent previously absent deficits that arise after surgery [6].

After a failed decompression procedure, patients can be grouped into three categories: persistent CTS, recurrent CTS, and new complaints. Careful clinical and neurological examination of the hand and upper extremity is essential to assess median nerve function in these patients [6]. Detailed evaluation, including Phalen and Tinel tests, as well as exclusion of polyneuropathies or systemic disorders, is critical for accurate diagnosis [6].

The most common cause of persistent symptoms is inadequate decompression of the median nerve at the carpal tunnel [4]. This is often confirmed intraoperatively as incomplete release of the flexor retinaculum. In overweight or obese patients, an insufficient incision length can leave the proximal or distal ends of the TCL unreleased [10,11]. Clinically, these patients present with a positive Phalen test and no evidence of compression elsewhere along the nerve [6].

Patients who initially experience symptom relief for six months or more before recurrence are more likely to suffer from secondary traction neuritis of the median nerve due to scar formation [12,13]. Incisions placed directly over the nerve predispose to adhesions between the median nerve, skin, and flexor retinaculum. Compared to persistent CTS, recurrent CTS is less common and often presents differently; patients more frequently report numbness and paresthesia rather than pain, and the typical nighttime paresthesia of primary CTS is usually absent [9].

An emerging treatment for persistent and recurrent CTS is complete median nerve release via external or internal neurolysis with soft tissue coverage [4]. The hypothenar fat pad flap technique mobilizes vascularized fat from the hypothenar eminence as a rotation flap, positioned between the neurolysed median nerve and the residual radial portion of the TCL. This flap cushions the nerve, reduces adhesion risk, and provides a smooth gliding surface. Flap tension is carefully assessed intraoperatively to avoid constriction, and early postoperative nerve gliding exercises are encouraged to maintain mobility and limit scar formation.

Strickland et al. emphasized that effective management of compression neuropathies requires relieving external pressure, preserving vascularity, and maintaining a functional gliding interface [14]. In their series of 58 patients treated with hypothenar fat pad flaps, 55 reported satisfaction, with 89% achieving relief from paresthesia and dysesthesia [14]. Both cases in the present report showed similarly favorable outcomes, with complete resolution of median nerve symptoms and no intra- or postoperative complications.

Nevertheless, these results should be interpreted with caution. The report includes only two cases, follow-up was short, and objective outcome measures beyond CTS-6 were limited. Thus, this study highlights a feasible treatment approach but does not provide definitive evidence of efficacy.

## Conclusions

External neurolysis with a hypothenar fat pad flap appears to be a reliable and relatively straightforward option for recurrent CTS. Optimal outcomes depend on meticulous surgical technique, careful intraoperative assessment of flap tension, and early rehabilitation. While these cases showed encouraging short-term results, larger studies with longer follow-up and standardized outcome measures are needed to establish efficacy.
